# The Journey of Cancer Cells to the Brain: Challenges and Opportunities

**DOI:** 10.3390/ijms24043854

**Published:** 2023-02-14

**Authors:** Marzena Łazarczyk, Michel Edwar Mickael, Dominik Skiba, Ewa Kurzejamska, Michał Ławiński, Jarosław Olav Horbańczuk, Jakub Radziszewski, Karolina Fraczek, Renata Wolinska, Justyna Paszkiewicz, Piotr Religa, Mariusz Sacharczuk

**Affiliations:** 1 Department of Experimental Genomics, Institute of Genetics and Animal Biotechnology, Polish Academy of Sciences, Postępu 36A, 05-552 Garbatka, Poland; 2 Department of Immunology, Genetics and Pathology, Uppsala University, SE-751 85 Uppsala, Sweden; 3 Department of Laboratory Medicine, Division of Pathology, Karolinska Institute, SE-141 86 Stockolm, Sweden; 4 Department of General Surgery, Gastroenterology and Oncology, Medical University of Warsaw, 02-091 Warsaw, Poland; 5 Institute of Genetics and Animal Biotechnology, Polish Academy of Sciences, Postępu 36A, 05-552 Garbatka, Poland; 6 Faculty of Medical and Health Sciences, Siedlce University of Natural Sciences and Humanities, Stanisława Konarskiego 2, 08-110 Siedlce, Poland; 7 Departament of Oncologic Surgery, Jan Paweł II Memorial Provincial Mazovia Hospital, 26, Księcia Józefa Poniatowskiego, 08-110 Siedlce, Poland; 8 Department of Pharmacodynamics, Faculty of Pharmacy, Medical University of Warsaw, Banacha 1B, 02-091 Warsaw, Poland; 9 Department of Health, John Paul II University of Applied Sciences in Biala Podlaska, Sidorska 95/97, 21-500 Biała Podlaska, Poland; 10 Department of Medicine, Karolinska Institute, SE-171 77 Solna, Sweden

**Keywords:** brain, metastasis, cancer, immune cells

## Abstract

Cancer metastases into the brain constitute one of the most severe, but not uncommon, manifestations of cancer progression. Several factors control how cancer cells interact with the brain to establish metastasis. These factors include mediators of signaling pathways participating in migration, infiltration of the blood–brain barrier, interaction with host cells (e.g., neurons, astrocytes), and the immune system. Development of novel therapies offers a glimpse of hope for increasing the diminutive life expectancy currently forecasted for patients suffering from brain metastasis. However, applying these treatment strategies has not been sufficiently effective. Therefore, there is a need for a better understanding of the metastasis process to uncover novel therapeutic targets. In this review, we follow the journey of various cancer cells from their primary location through the diverse processes that they undergo to colonize the brain. These processes include EMT, intravasation, extravasation, and infiltration of the blood–brain barrier, ending up with colonization and angiogenesis. In each phase, we focus on the pathways engaging molecules that potentially could be drug target candidates.

## 1. Introduction

Brain metastasis (BM) is the most common type of intracranial neoplasm in adults. In addition to end-of-life status, brain metastasis patients suffer from various distressing symptoms such as headache, cognitive impairment, ataxia, and seizures. Secondary brain tumors are metastases to the brain originating from primary tumors located outside the central nervous system (CNS). They occur more frequently than primary brain tumors. It has been revealed that the incidence of BM doubled between 1987 and 2006 [1]. The increase in BM incidence is due to a combination of different factors. These variables include improvement in the detection rate of lesions upon advancements in diagnostics and successful treatment modalities resulting in longer survival following the diagnosis of primary cancer. However, the prognosis of brain metastasis is currently devastating [2]. The median survival for BM patients is between 5 weeks and 18 months. Several factors control the length of the survival of BM patients. These factors include: the source of the primary tumor, the number of metastatic foci in the brain, the treatment method, and the BM location.

(1) There is a slight variation among BM patients based on the source of the primary cancer (Table 1). Notably, it was reported that lung cancer is responsible for 80% of reported BM cases, while melanoma, breast cancer, and kidney cancer are responsible for 3.8, 3.7, and 3% of the BM cases, respectively [2,3]. However, in another study, it was reported that secondary brain tumors are most likely to derive from breast cancer, followed by lung carcinomas, renal cell carcinomas, and melanomas [4]. The reasons behind these discrepancies could be related to the age of patients, for example, it was shown that kidney/renal tumors and melanomas are the most frequent in children [3]. Gender could also play a significant role in determining the origin of the BMs cells; it was reported that the primary cancers most often responsible for BM are of the breast in women and of the lung in both genders (Table 1);
(2) The number of metastatic foci has been shown to be inversely proportional to prognosis, with a worse outcome predicted based on the detection of one or more metastatic sites [5]. Due to constant improvements in diagnostic and therapeutic strategies, the median survival among patients with metastatic disease of the brain has been reported to have increased [6];
(3) The type of treatment influences the survival rate, as it has been shown that patients undergoing specified therapy, including surgery, chemotherapy, or radiotherapy, have a higher chance of surviving longer [7];
(4) Further investigation is needed to pinpoint the relationship between BM location and survival. Patients suffering from BMs in the frontal lobe have a poorer prognosis compared to those with non-frontal lobe BMs (4.9 and 10 months, respectively). Furthermore, metastasis infiltrating the eloquent cortex (containing speech and sensory fields) in relation to non-eloquent areas is associated with higher median survival (21.2 months vs. 18 months). Interestingly, several studies have revealed that 85% of metastasis cases are reported in the cerebrum, including the posterior areas of the two hemispheres and the anterior border zone between the anterior and middle cerebral arteries. Conversely, only 10% to 15% of metastasis cases are detected in the cerebellum, followed by 3% in the brainstem. However, studies comparing the survival of patients based on the BM locations in these regions seem to be scarce [8,9].

## 2. The Metastatic Cells Journey to the Brain

**Figure 1:**
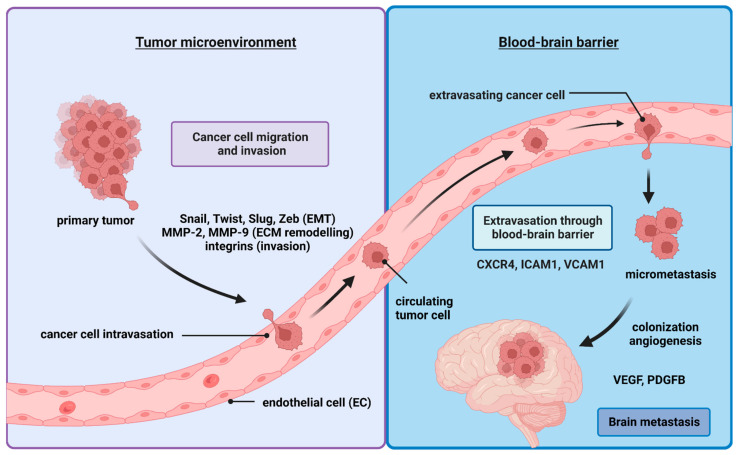
The major stages of metastasis formation in the brain. Metastatic cells pass through several phases in order to colonize the brain. First, they acquire a stem-cell-like phenotype and shed their epithelial phenotype in a process called epithelial–mesenchymal transition (EMT). Once the EMT phase has started, cancer cells begin degrading the ECM and reshaping it to form a hole the approximate size of one cancer cell. After that, they have to choose which intravasation passage they take. There is a need for better understanding of the factors affecting cancer cells’ migration routes. Nevertheless, after finishing the intravasation phase, the cancer cells initiate the extravasation phase, where they infiltrate the blood–brain barrier (BBB), and, finally, they reverse their status from stem-cell-like back to the epithelial-like phenotype as they start to colonize the brain. The interaction between cancer cells and their environment constitutes a large mine of therapeutic opportunities aimed at halting cancer cells’ invasion of the brain. Created with Biorender.com.

The molecular course of BM development follows various stages (Figure 1). The metastatic dissemination starts with phenotypic changes of the tumor cell and detachment from the primary lesion, followed by invasion of the extracellular matrix (ECM), intravasation into blood or lymphatic vessels in the form of circulating tumor cells (CTCs), spreading via the circulation or the lymphatic system, extravasation, and colonization. To survive, cancer cells have to overcome obstacles, including susceptibility to apoptosis or anoikis (resulting from detachment from ECM and neighboring cells), physical forces related to blood flow, and several types of immune cells, such as microglia, macrophages, CD4+ T cells, CD8+ T cells, and natural killer T cells. In this review, we cover the stages that cancer cells follow to migrate from the primary site to their secondary residence in the brain. We also discuss current knowledge, addressing how these cells challenge their surroundings.

### 2.1. Epithelial–Mesenchymal Transition (EMT)

Cancer cells preparing to invade the surrounding tissue as their first step of the metastatic journey undergo epithelial–mesenchymal transition (EMT) [10,11]. This complex process constitutes cells shedding their epithelial phenotype to become less differentiated, more aggressive, and more stem-cell-like [12]. To perform this crucial transition, cancer cells utilize a large group of genes and transcription factor networks. These transcription factors include: Slug, Twist, Snail, ZEB1, and ZEB2. These proteins are activated in response to signaling cascades, regulated by transforming growth factor β (TGF-β), bone morphogenetic protein (BMP), epidermal growth factor (EGF), fibroblast growth factor (FGF), and platelet-derived growth factor (PDGF), as well as other signaling molecules (e.g., Wnt, Shh, Notch, and integrins) [13]. Overall, these genetic networks collaborate to acquire various mesenchymal properties, such as increased cytoskeletal reorganization and decreased cell adhesion. Notably, during EMT, cancer cells use several strategies to protect themselves against destruction by immune cells.

Scaffold protein rearrangement takes place through the upregulation of the expression of mesenchymal cell markers (e.g., type III intermediate filament vimentin). During EMT, the vimentin network not only plays a role as a passive scaffold for organelles in the cytoplasm, but also displays an active commitment to the EMT-related signaling pathways. It has been shown that vimentin supports the extracellular signal-regulated kinase (ERK)-mediated phosphorylation of the Slug transcription factor, which actively participates in EMT [14,15]. To date, different molecules have been identified that are involved in EMT of primary cancer cells and migration to the brain. Metastasis-related lung adenocarcinoma transcript 1 (MALAT1) is a regulatory long non-coding RNA. MALAT1 has been shown to regulate over two hundred genes. One of its main functions is supporting cancer cell migration through targeting the Rho/ROCK signaling pathway [16,17,18,19]. MALAT1 can also induce transcription factors, such as ZEB1, leading to TGF-β-mediated EMT in human keratinocytes [20]. In patients with non-small-cell lung carcinoma (NSCLC), brain metastases and poor prognosis are associated with the presence of MALAT1 in primary tumor samples. MALAT1 has been demonstrated to change the differentiated cobblestone-like shape of epithelial cells into a spindle-like mesenchymal phenotype with increased motility, promoting EMT and brain metastasis [21]. Furthermore, MALAT1 silencing in highly invasive human lung cancer H1915 cells prevented brain metastasis formation in athymic BALB/c mice compared to control animals. Interestingly, in 2018, a report appeared highlighting an anti-metastatic effect of MALAT1 in breast cancer [22]. Therefore, the same molecule may demonstrate opposite activities depending on primary tumor type.

Cells transitioning into a mesenchymal phenotype utilize multiple genes. One of them is cell adhesion molecule 2 (CADM2), used by cancer cells in both cytoskeletal organization and cell adhesion since it plays a fundamental role in the aggregation and forming of cell clusters. This adhesion molecule, under normal conditions, modulates axonal myelination in oligodendrocytes and participates in the arrangement of synapses [23]. In NSCLC patients with brain metastases, CADM2 was upregulated over twofold as compared to in non-metastatic NSCLC patients. The silencing of CADM2 in several NSCLC cell lines (e.g., A549 and H322) resulted in lower vimentin levels, accompanied by a decrease in cell migratory ability and an increase in the expression of epithelial marker E-cadherin. Hence, CADM2 both induces EMT and supports migration of lung cancer cells to the central nervous system. Cells undergoing EMT also upregulate N-cadherins but downregulate expression of E-cadherins and the epithelial cell adhesion molecule (EpCAM) [24]. One of the regulators of E-cadherin is SNORA17B. SNORA17B belongs to snoRNAs, non-coding RNAs, and is involved in ribosomal RNA methylation. SNORA17B is highly expressed in the brain metastasis of breast cancer patients and correlated with poorer prognosis [25]. SNORA17B-transfected human breast cancer cell lines show higher invasion ability and reduced E-cadherin expression. Thus, it is postulated that SNORA17B directly/indirectly controls E-cadherin levels. Another gene associated with cell adhesion is human epidermal growth factor receptor 2 (HER2). HER2 is a transmembrane protein with tyrosine kinase activity. HER2 promotes phosphorylation reactions, leading to the initiation of several signaling pathways mediated by STAT3, RAS-MAPK, which results in the inhibition of pro-apoptotic proteins and an increase in the expression of cell-proliferation-related genes, supporting EMT and metastasis to the brain [26,27]. HER2 has been found to be frequently co-expressed with junctional adhesion molecule-A (JAM-A) in aggressive breast cancer, where it participates in tight junction formation in epithelial/endothelial cells [28,29]. Recently, HER2-overexpressing human breast cancer cells have been evidenced to induce greater expression of Snail, Slug, and ZEB1 transcription factors, greater TGF-β production, and increased level of N-cadherins, followed by a decrease in expression of E-cadherin and cytokeratin-18 [30]. HER2 could constitute one of the main drug targets for designing therapies to inhibit metastasis, as it was shown that HER2 inhibition reduced metastases not only to the brain but also to lungs and liver in nude mice by 86%, 50%, and 64%, respectively [30]. Another molecule linking EMT with brain metastasis is astrocyte elevated gene-1 (AEG-1). Its overexpression entails upregulation of N-cadherin and Slug but reduction in expression of epithelial markers E-cadherin and ZO-1 (zonula occludens-1). AEG-1 is an endoplasmic reticulum (ER)-associated cytoplasmic RNA binding protein, interacting with numerous mRNAs encoding secretory, cytosolic, and organelle proteins [31]. AEG-1 was identified for the first time in primary astrocytes of the human fetus, localized predominantly in the endoplasmic reticulum but also near the cell nucleus. In time, this oncogene was found in the cell membrane of breast cancer cells. Functions that were assigned to AEG-1 in cancer include: malignant transformation, resistance to chemotherapy (being a co-inducer of chemoresistance-associated genes), and anoikis, angiogenesis, and metastatic spread [32,33,34].

EMT is frequently associated with the downregulation of β-catenin, involved in creating cell junctions [35]. Of note, metastatic cancer cells downregulate several other genes involved in forming cell–cell connections, such as those encoding for desmoplakins (building desmosomes) or proteins responsible for providing the cell with the resistance to mechanical stress such as cytokeratins [36,37].

Cancer cells, in order to survive, have to protect themselves from the effect of immune cells. It has been shown that cancer cells deplete tryptophan in their microenvironment by producing indoleamine 2,3-dioxygenase (IDO), which metabolizes tryptophan to kynurenine. Tryptophan deficiency causes a T-cells-mediated stress response, which prevents them from proliferating. Kynurenine induces the development of regulatory T cells (Tregs) and enhances the production of anti-inflammatory cytokines, such as IL-10 and TGF-β [2,38].

### 2.2. Cancer Cell Infiltration of the ECM

One of the first obstacles that migrating cells face is the extracellular matrix (ECM). For cancer cells to overcome this powerful barrier, they follow several phases. First, they form invadopodia, which are transformed into a pseudopod protrusion, followed by the creation of focal contacts, local proteolysis, actomyosin contraction, and, finally, the detachment of the trailing edge [39,40].

Tumor cells start their journey as invadopodia. One of the main characteristics of invadopodia is the high enrichment of fast-polymerizing actin (F-actin) filaments [41]. Src family kinases and their substrates play a pivotal role in the actin dynamics of the invadopodia and pseudopodia. For example, Tks5 acts as a protein scaffold. Another Src partner, a cross-linking protein, actin-filament-associated protein of 110 kDa (AFAP110), modulates actin filament integrity [42]. Recent reports have indicated that the Src-dependent regulatory mechanisms are mediated by several other pathways such as reactive oxygen species and microRNA [43,44].

**Figure 2:**
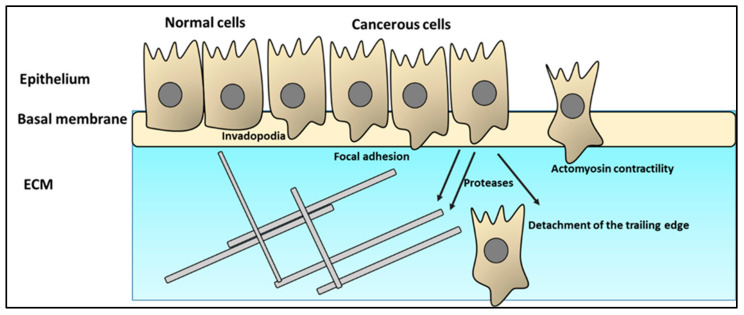
Cancer cell infiltration of the ECM. The process constitutes several phases such as (1) pseudopod protrusion formation, (2) construction of focal contacts, (3) focalized proteolysis, (4) actomyosin contraction, and, finally, (5) the detachment of the trailing edge.

Focal adhesions (FAs) are sites of integrin clustering that link the actin cytoskeleton to the ECM. The primary function of FAs is to provide physical attachment to the ECM and transduce force between the cell and the ECM (Figure 2). The FA component, a focal adhesion kinase (FAK), regulates FA signaling by providing a scaffolding function for the protein–protein interactions and regulating the cross-talk between integrins and growth factor signaling [45]. Proteolysis of the ECM via recruitment of surface proteases is the primary goal of this phase. Proteases such as MT1-MMP, MMP2, MMP9, ADAM12, ADAM15, ADAM19, cathepsins, and seprase usually accumulate in the invadopodia [46]. These enzymes, once secreted, contribute to localized pericellular degradation of proteins. In cancer cells, genes encoding metalloproteinases ae usually upregulated. The main aim of this step is to degrade the extracellular matrix. Interestingly, cancer cells can also exploit normal fibroblasts to produce significant amounts of MMP2 [47,48]. Secondary remodeling via ECM component secretion results in realigning ECM fibers to remain stable in a new form that contains a hollow tube the size of the cell’s diameter [49].

Actomyosin contractility promotes cancer cell colonization and outgrowth [50]. Contractility is under the control of a wide variety of pathways, including SRF/MRTF, TGF-β-SMAD-CITED1, MMP-9, BRAF-V600E, and CDC42 signaling [50]. Similarly, it has been speculated that detachment of the trailing edge is calcium dependent. Notably, it has been shown that various extracellular matrix (ECM) components play a role in protecting migrating cancer cells through preventing antigen presentation to T cells [51]. Fibroblasts produce matrix metalloproteases (MMPs) to degrade the ECM [52]. Mesenchymal stromal cells produce TGF-β to weaken the immune response. Necrosis caused by ECM disruption by cancer cells induces myeloid-derived suppressor cells (MDSCs) to downregulate the antitumor immune response by degrading various amino acids in the tumor microenvironment. Under such conditions, T cells starve, and the signaling pathways required for their activation are inhibited. Another pathway that MDSCs utilize to suppress T cell proliferation and activation is the production of nitric oxide (NO), which hinders IL-2 signaling. MDSCs produce reactive oxygen species (ROS), which increase T cell apoptosis. ROS can combine with NO to generate peroxynitrite, which disrupts the TCR–MHC interaction, resulting in increased cancer cell resistance to cytotoxic T cell responses [53].

### 2.3. Intravasation

During their migration to the brain, cancer cells can either use blood circulation or the lymphatic vessels pathway [54,55]. The role of the lymphatic system in metastatic spread has been recently reported in detail. In an interesting experiment, mice were injected with murine 4T1 breast cancer cells via lymphatic vessels without prior inducement of a primary tumor [56]. Notably, tumor cells were detected close to the blood vessels two days post inoculation, and, on the third day, the cells had entered the blood circulation, mainly through postcapillary gates named “high endothelial venules”. Importantly, the presence of cancer cells, in the form of CTCs, was also detectable in the blood on day 3 following injection. After 35 days, the cancer cells colonized lungs in a similar time–space pattern of dissemination to that achieved upon orthotopic administration. Therefore, blood vessels of lymphatic nodes constitute an effective entry point for tumor cells to access circulation [56]. The factors that govern the decision of the cancer cells to migrate through the blood vessels, or the lymphatic vessels, are still being investigated; however, certain prerequisites have been already determined. They include:

(i) Differences in function between blood vessels and lymphatic vessels. The lymphatic system’s main functions include transferring various macromolecules, fluids, and immune cells to the blood and maintaining plasma volume [57]. The composition of the lymph fluid is almost identical to that of the interstitial tissue fluids, which promote the survival of migrating tumor cells [55];
(ii) Blood and lymphatic vessels differ considerably in their structure [55]. While blood circulation constitutes a closed system, the lymphatic system flows in one direction from the peripheral tissues to the blood. To achieve its function, the lymphatic system uses lymphatic capillaries and pre-collectors, followed by lymphatic vessels and trunks that reach the bloodstream. Lymphatic vessels are threefold the size of blood vessels, with an incomplete, discontinuous basal lamina, absence of pericytes, and smooth muscles. These differences in structure mean that the migrating cells face lower mechanical resistance in traveling through lymphatic vessels compared to through blood vessels. Thus, less energy is needed by the cancer cells to travel through lymphatic vessels. Lymphatic vessels are leakier, thus, supporting cancer cells to spread more [55,58];
(iii) The role of ECM: cancer cells migrating through the blood and lymphatic vessels use ECM to their advantage. Endostatin, tumstatin, canstatin, arresten, hexastatin, and type IV and type XVIII ECM collagens all have a significant influence on the creation of both blood and lymphatic vessels [55,59]. Integrin 91, an ECM receptor, has been linked to the development of lymphangiogenesis [60]. Similarly, low-molecular-weight hyaluronan promotes lymphangiogenesis by interacting with its lymphatic vessel endothelial hyaluronan receptor 1 (LYVE-1), promoting lymphatic endothelial cell proliferation and tube formation. Lymphangiogenesis and tumor invasion are tightly connected to MT1-MMP-mediated proMMP-2 activation and the production of ECM1 and EMILIN1, an elastic microfibril-associated protein [61,62];
(iv) Expression of various genes and receptors specific to blood or lymphatic vessels, as well as cross-talk between lymphatic and blood vessels, could also contribute to the decision of cancer cells when choosing their route.

After infiltrating the lymphatic or blood vessel wall, tumor cells are known as circulating tumor cells (CTCs). Notably, a small portion of CTCs survives in the bloodstream and forms metastasis. There are several stress factors CTCs face in the blood, including: hemodynamical forces, anoikis (e.g., programmed cell death upon loss of contact with ECM), and encountering immune cells, as well as narrow microcapillares posing a risk of deformation or of being entrapped. Hemodynamical forces may trigger mechanical damage of the cell. Two essential, biomechanical forces act on the vascular wall: tensile stress resulting from blood pressure and shear stress as tangential (parallel) force to the endothelial cells surface. Additionally, turbulent blood flow at arterial bifurcations, collisions with vascular wall, and other morphotic elements of blood are all noxious factors that may result in CTC deformation, damage, apoptosis, or death [63].

To avoid anoikis, CTCs form clusters that display higher metastatic potential than single cells [64,65]. The third obstacle CTCs must overcome is immune cells, particularly natural killers (NK), which are able to lyse cell intruders [66,67]. CTCs also express tissue factor (TF) on their surface. TF activates the coagulation process and formation of platelets-rich envelope around CTCs [66]. Within such a coating, CTCs form a connection with thrombocytes via integrins, and such a barrier is considered to protect CTCs from shear stress or NK cells [68,69]. Importantly, coagulation and thrombocyte activation are known mechanisms that accelerate metastases formation [70,71]. By producing TGF-β and downregulating NKG2D on NK cells, platelets also inhibit natural killer cells [72]. Platelets can provide normal MHC class I molecules to tumor cell surfaces, shielding them from cytotoxic T cells [73]. One explanation for how macrophages boost CTC survival is the production of α4-integrin, which interacts with VCAM-1 on the surface of CTCs and provides a survival signal [74].

### 2.4. Extravasation through the Blood–Brain Barrier

The next stage of cancer cells’ invasion of the brain is infiltration of the blood–brain barrier (BBB). The BBB is a complex barrier formed of endothelial cells, astrocytes, basal membranes, and pericytes. During their attempt to cross the BBB, cancer cells aim to exploit the surrounding microenvironment. Cancer cells attack leukocytes and regulate their production of various cytokines to facilitate their infiltration of the brain [75]. Chemokines play a central role in directing cancer cells toward the endothelial cells’ surface and guiding the process of infiltration of the brain. To be able to home to the endothelial cells of the brain, cancer cells have been shown to express CXCR4 chemokine receptors, which can increase the chemotaxis attraction of cancer cells to the BBB [76]. Notably, cancer cells seem to enter a state of arrest, with no recorded proliferation during the time preceding the infiltration [77,78]. The morphology of the cancer cells changes based on their location within the extravasation process. At first, while in the arrest state, they take an elongated shape. After that, before the start of the transmigration process, they acquire a round morphology, in which they exert pressure on the vessel walls. In an in vitro study by Abidine et al., J82 cancer cells intending to transmigrate through the endothelial monolayer had a round shape with intracellular actin localized at the periphery, followed by an ovoid morphology with actin gathered near the endothelial gap [79].

The infiltration process includes three main stages, namely, rolling, adhesion, and transmigration. This process seems similar to that of leukocyte infiltration of the brain, albeit less well described. Interestingly, it has been found that the time needed for extravasation is cancer type dependent. For example, lung cancer cells were reported to take around two days to infiltrate the brain, while breast cancer cells were shown to need between two and seven days [80]. During the transmigration process, the cancer cells’ morphology exhibits a flexible structure, with parts of the cells outside and other parts within the vascular wall. Additionally, they show changes in their cellular extensions, showing a dynamic interaction between invasive cancer cells and endothelial cells [81,82].

The rolling process seems to depend on selectins. Cancer cells express selectin ligands, which mediate the tethering and rolling of cancer cells on the surface of the endothelial cells. They also can be used to adhere to leukocytes, which, in turn, support the attachment of cancer cells to the vessel walls. Heparin inhibits selectin-mediated cell adhesion, which slows the process of extravasation of melanoma, confirming a significant contribution of selectins to the success of the rolling process [83,84].

Once the rolling stage has ended, cancer cells begin binding to the endothelial cells. Endothelial cells express intercellular adhesion molecule 1 (ICAM-1) and VCAM-1, which are important in the attachment between cancer cells and the endothelial cells, as demonstrated on a lung cancer model [85]. Similarly, cadherins, including E and N subtypes, have been shown to contribute to lung cells’ interaction with endothelial cells of the BBB [86]. Other genes that seem to play a role in the interaction between cancer cells and endothelial cells include endothelial cell receptors as integrins. Inhibition of endothelial cell receptors results in inhibition of the interaction between invading cancer cells and the endothelial cells and reduction of brain metastasis. Integrins interact with ECM components such as collagen, laminin, and fibronectin. Integrins can also mediate the production of cells’ survival signals. One of the main integrins that has been demonstrated to be expressed by lung metastatic cancer cells in the brain is A3b1. A3b1 inhibition resulted in a decrease in brain metastases in nude mice [87]. However, all the pathways controlling the adhesion process are yet to be determined.

**Figure 3:**
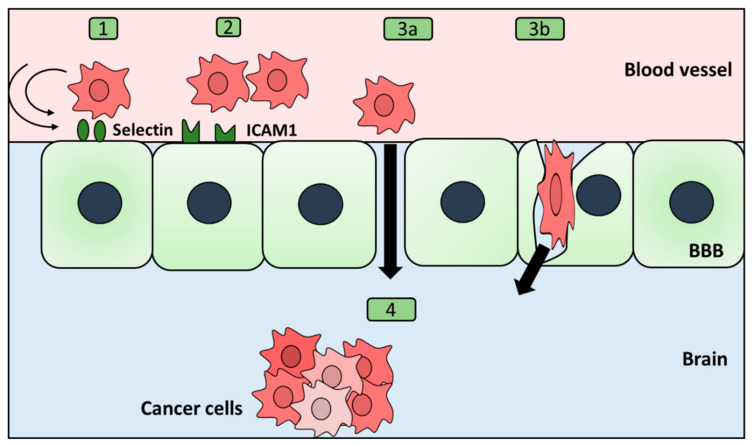
Cancer cells infiltrating the blood–brain barrier. The infiltration process comprises three primary phases: (1) rolling, (2) adhesion, and (3a) transmigration. Transmigration can take a paracellular route (between the cells) or (3b) transcellular route (through the cell). (4) After infiltration of the BBB, cancer cells choose either to colonize the areas proximal to endothelial cells or to migrate further into the brain.

Interestingly, it has been shown that metastatic cancer cells have two main choices of transmigration, namely, (i) paracellular transmigration and (ii) transcellular transmigration, in a way similar to leukocyte transmigration mechanisms (Figure 3) [78,88]. For example, melanoma cells use a paracellular route, while breast cancer cells follow a transcellular route [78]. MMPs are particularly important for paracellular transmigration because of their role in degrading junctions between cells [89]. Another critical alteration by the migrating cancer cells is rho/rho kinase signaling, which has been shown to support paracellular infiltration of the brain [90]. In the case of the paracellular route, downregulation of genes responsible for stabilizing BBB integrity, such as β-catenin and zonula occludens (ZO)-1, as well as a gap junction protein—connexin 43 (CX43), has been reported [91]. Furthermore, rearrangements of the cytoskeleton have also been observed through increasing myosin light chain kinase (MLCK) and phosphorylated myosin light chain (p-MLC), resulting in endothelial cell contraction [92]. On the other hand, in the case of transcellular migration, caveolin 1 (CAV1) levels are upregulated [91]. One molecule that may play a role in the specificity of brain metastasis is phosphatidylinositol 3-kinase (PI3K). It has been shown that its inhibition leads to a reduction in metastasis formation of breast cancer but not of lung cancer. Whether it plays a role in controlling the transmigration route is still to be determined. Additionally, the complete map of genes determining the transmigration route is yet to be drawn. Interestingly, after finishing the extravasation process, breast cancer cells favor localizing in proximity to the endothelium and delay the formation of their own vasculature [93]. Conversely, melanoma or lung cancer cells seem to favor switching early angiogenesis and migrating further into the brain [81]. The reason behind their choices remains speculative.

BBB permeability

Various reports suggest that the BBB is often compromised during brain metastasis. Tumor cells aim to increase leakage of the BBB. One of the mechanisms they use is to alter the lipid metabolism by inhibiting the expression of the endothelial cell fatty acid transporter Mfsd2a. Conversely, some strategies for disrupting the BBB to enhance drug delivery to the brain have been reported [94]. The treatment of CNS tumors by radiation often results in increased BBB permeability [95]. Cytostatics used in breast cancer therapy, such as taxane docetaxel, can disrupt the BBB. Taxane docetaxel usage is correlated with an increase in brain metastasis [96]. Thus, the strategy of increasing brain permeability could need to be tuned based on the individual patient’s health condition, the type of cancer, and the stage of the disease.

### 2.5. Mesenchymal-to-Epithelial Transition (MET)

**Figure 4:**
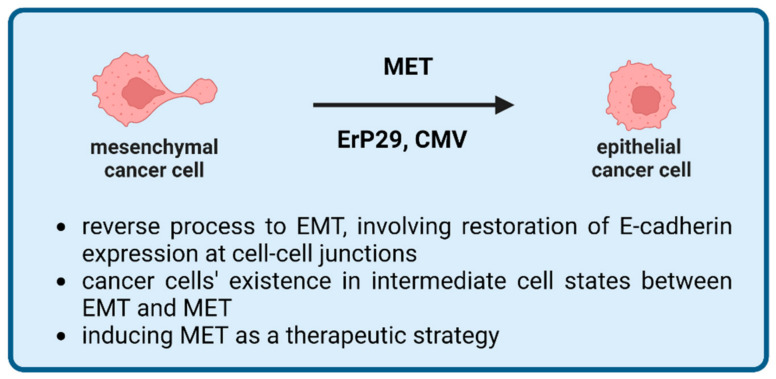
Mesenchymal-to-epithelial transition (MET) is the reverse process to EMT. In MET, mesenchymal cells gain epithelial traits. MET can be considered as an early stage of adaptation for CTCs to a new environment, and its induction has been discussed as a potential therapeutic strategy. MET involves numerous genes/proteins and other external factors including, but not limited to, ErP29 or CMV. Created with Biorender.com.

Mesenchymal-to-epithelial transition is a reprogramming process that aims to reverse the process of EMT (Figure 4) [97]. MET could be considered an early stage of the adaptation of a CTC to its novel conditions (colonization). The MET transition is characterized by the re-expression of the genes that were silenced during EMT, followed by changes in the cancer cell morphology, which have been confirmed experimentally [98]. One of the hallmarks of MET is the restoration of E-cadherin expression at cell–cell junctions [99]. To achieve this, intracellular domains of E-cadherin bind to the cytoskeleton through linking “bridges” such as β-catenin [100]. This structure modulates the interactions between cells and ensures epithelial phenotype maintenance. Interestingly, investigators exploring metastatic tissues uncovered that tumor cells can exist in an intermediate state between EMT and MET [101]. This transient phase of cellular reprogramming involves both apparent co-expression of the epithelial marker such as E-cadherin and mesenchymal markers such as vimentin. These situations seem to take place regardless of the primary cell type [102]. Molecular reprogramming requires a switch in expression of many other genes followed by intense metabolic processes such as protein synthesis, intracellular transport, and secretion. Endoplasmic reticulum (ER) protein 29 (ERp29) is highly expressed throughout mammalian tissues. It predominantly acts as an ER to Golgi escort/chaperone proteins [103,104,105,106]. Cancer research revealed additional properties of ERp29, including cell growth arrest and involvement in MET. ERp29-transfected human breast cancer MDA-MB-231 cells have been demonstrated to change the morphology into one that is more epithelial and to stimulate growth in clusters, while control cells retained their predilection for more scattered growth in vitro. When injected into nude mice, breast cancer cells overexpressing Erp29 demonstrated delayed tumor formation as compared to control [107].

In addition to the endogenous, intracellular mechanisms controlling MET, some external factors may contribute to the transition towards the epithelial phenotype. A few years ago, it was discovered that, in a response to human cytomegalovirus (HCMV) infection, breast carcinoma and glioma stem cells inhibit EMT and induce MET [108]. The question of whether HCMV supports colonization of metastatic cells, or, rather, renders them less malignant, is still open. It is, however, possible that CMV realizes the first scenario, as shown by earlier reported evidence indicating the virus entanglement in enhancing the metastatic spread [109,110]. Induction of the MET state seems to have some therapeutic implications in oncology. Targeted and coerced transformation of malignant cells into less proliferative and migratory ones is an attractive approach. First attempts to reverse EMT have been already made, for example, on human mesenchymal mammary epithelial cells. Exposing them on a cholera toxin or forskolin induced an increase in the intracellular levels of adenosine 3′,5′-monophosphate (AMP), followed by an activation of protein kinase A (PKA). As a result, mesenchymal-to-epithelial transition was observed in the treated culture [111]. Reaching primary tumors or metastasis in the brain with drugs that are able to change phenotype selectively appears to be challenging but is worth further exploration.

### 2.6. Angiogenesis

**Figure 5:**
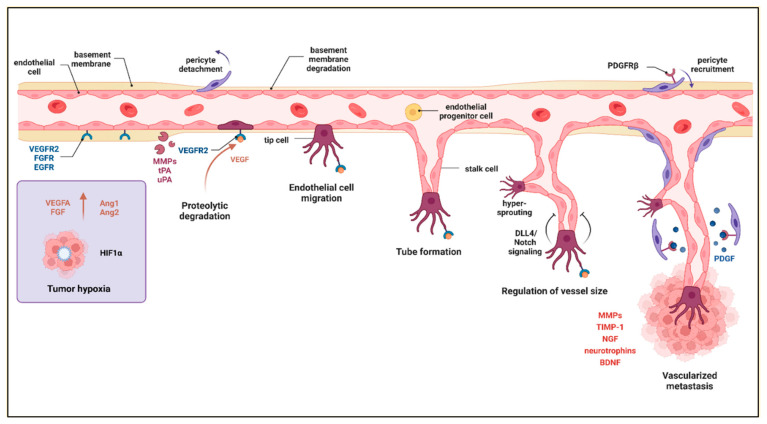
Tumor angiogenesis. To ensure the success of new blood vessel formation, a cascade of events usually occurs, including: hypoxia, proteolytic degradation, endothelial cell migration, tube formation, regulation of vessel size, and formation of tumor vasculature. Created with Biorender.com.

Angiogenesis refers to the sprouting of new vessels from preexisting ones. Both primary and secondary tumors need constant delivery of nutrients and oxygen for growth and expansion. However, at the initial stages of their development, vasculature construction is less frequently observed. The vascular network density usually correlates with tumor grade. The higher number of formed microvessels in the cancer lesion, the more advanced the disease [112]. Migration of endothelial cells is essential for new vessel creation, as well as their communication with ECM. These interactions are provided by ECM fibronectins and endothelial integrins, among others [113]. The importance of integrins in the angiogenesis of brain metastasis was demonstrated in SCID mice inoculated with human MDA-MB-435 breast cancer cells with activated integrin αvβ3 [114]. As a consequence, instead of tumor growth in the primary site (mammary pad), metastatic spread to the brain was observed. The mechanism involved activated integrin αvβ3-induced translation of VEGF mRNA [114]. To prepare the space for the developing tumor vasculature, ECM decomposition was initiated through tumor cells releasing proteases activating plasmin (tissue-type plasminogen activator and urokinase-type plasminogen activator) and, subsequently, MMPs [115,116]. Several other molecules involved in angiogenesis have been described, including vascular endothelial growth factor (VEGF), fibroblast growth factors (FGFs), angiopoietin-1 (Ang1), and angiopoietin-2 (Ang2), acting through the Tie2 receptors, interleukin-8, matrix metalloproteinase-2, and many more (Figure 5) [117]. It has been shown that VEGF is highly pro-angiogenic, especially under limited availability of oxygen [118]. The structure of tumor vessels is fragile, with increased permeability and a tendency to collapse, which results in incidences of local hypoxia followed by necrotic core development. Oxygen deficiency is a strong accelerator of angiogenesis and cancer progression. One of the mechanisms induced by lack of oxygen is the increase in the cellular level of hypoxia-inducible factor 1 (HIF-1). HIF-1 activates the expression of various genes related to angiogenesis such as angiopoietin 1 and 2, matrix metalloproteinase 2 and 9 (MMP-2 and -9), platelet-derived growth factor B (PDGFB), and vascular endothelial growth factor A (VEGFA) [119]. Recurrent tumor vessel collapses and repeated hypoxia may lead to the continued release of VEGF by cancer cells and angiogenesis. The described process, seen in gliomas, is an example of how a tumor supports its growth in an autocrine manner [120]. Together with the increase in VEGF at more advanced cancer stages, a rise in the levels of regulatory molecules takes place. These mediators modify the expression of potent, pro-angiogenic factors. Among them are microRNAs that may function as oncogenes or gene suppressors. In relation to secondary brain tumors, for instance, miR-378 has been evidenced to be upregulated in thoracic specimens of the patients with NSCLC and brain metastases as compared to in NSCLC cases without metastatic spread in the CNS [121]. Further in vitro and in vivo experiments confirmed that miR-378 binds to VEGF 3′ UTR and promotes VEGF expression [122]. Considering HIF-1α, examination of clinical specimens from patients with lung, breast, kidney, colorectal, and melanoma cancers revealed the highest expression level and microvascular density in brain metastasis of renal cell carcinomas, but the lowest in the metastatic lesion in the CNS of the melanoma patients [123]. Simultaneously, HIF-1α expression was significantly higher in brain metastases, than in matched primary tumors of patients with breast cancer, non-small cell lung carcinoma and colorectal cancer [123]. These observations are in line with another investigation, which evidenced that brain metastasis of melanomas demonstrates lower tumor microvasculature counts than breast and lung carcinomas [124]. The extent of vasculature formation in metastases depends on various factors, such as primary cancer type, disease stage, and the specific growth rate of given tumors. In the course of cancer invasion/metastasis, balanced action of both matrix metalloproteinases (MMPs) and tissue inhibitors of matrix metalloproteinases (TIMPs) is required to provide both vascular remodeling and prevention of structural abnormalities due to extensive outgrowth, respectively [125]. Interestingly, implantation of human lung cancer cells or their TIMP-1-overexpressing counterparts into the brain of nude mice resulted in the development of larger tumors along with a twofold increase in the number of vessels in the latter group as compared to the control [126]. These results are consistent with in vitro studies on human umbilical vascular endothelial cell (HUVEC) cultures growing in the presence of a higher concentration of TIMP-1, which enhances tubule formation [127,128]. It seems that the cited in vivo study is an example of the beneficial effect of inhibition of ECM degradation for tumor progression, probably due to reduced vascular malformations. Last but not least, an aspect of angiogenesis in brain metastases is the specific microenvironment being rich in neuronal- (or glial)-derived trophic factors, including nerve growth factor, neurotrophin-3, neurotrophin-4, and brain-derived neurotrophic factor (BDNF), binding to different Trk kinases [129]. For example, BDNF was found to induce VEGF expression in neuroblastoma cells upon interaction with TrkB and downstream activation of HIF-1α [130].

## 3. Survival of Cancer Cells in the Metastatic Environment

Migratory CTCs must adapt to the distinct microenvironment to survive and colonize, forming metastasis. Further spread of metastatic cells following colonization requires extracellular matrix remodeling [131]. After BBB invasion, it is hypothesized that these cells initially proliferate along the microvasculature rather than into the brain parenchyma (a tendency described as “vascular cooption”). This is necessary to establish microcolonies [132]. In the brain, metastatic cancer cells face the opposition of the glia, microglia, and neurons counteracting the penetrating intruders. However, the effect of the response of the host may not always be favorable.

Cancer cells are capable of exploiting neurons to achieve their goal of colonization. To ensure their glutamate supply, breast cancer cells push out and replace astrocytes in the tripartite synapses, forming pseudo-tripartite synapses [133]. This process ensures NMDAR (N-methyl-D-aspartate receptors) activation, which, in turn, supports cell division. NMDA receptor antagonists, such as MK-801 (dizocilpine), have been shown to possess anti-proliferative and anti-invasive effects [134]. Perplexingly, it was shown that NMDAR activation using glycine_B_ site ligands also results in anti-proliferative effects [133,135]. Another dimension of complexity arises from the ability of metastatic cells to alter their phenotype. This change includes the ability to produce GABA-associated proteins such as GABA transaminase (ABAT), GABA A receptor (GABA A R), glutamate decarboxylase (GAD67), GABA transporter, etc. [136]. In this sense, cancer cells utilize GABA as a source of energy by metabolizing GABA into succinate. This was demonstrated by the observation that inhibiting GABA uptake in metastatic cancer cells decreases NADH levels and limits their proliferative activity [136].

**Figure 6:**
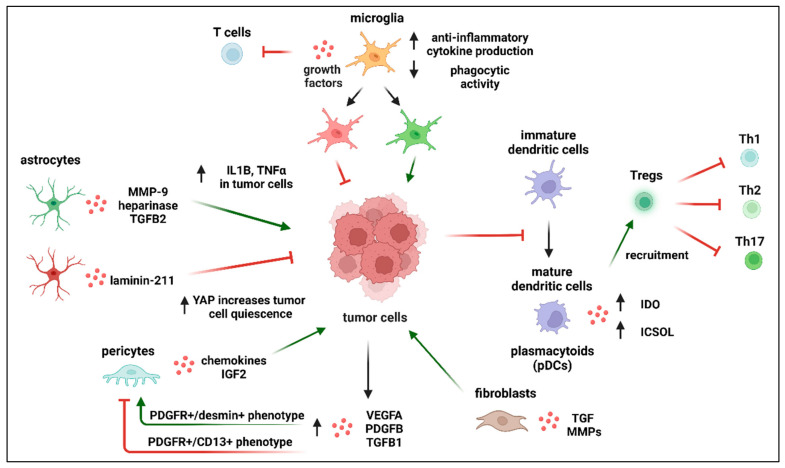
Metastatic cell interactions with their microenvironment. Astrocytes can inhibit the growth of cancer cells through the production of laminin 211, which increases YAP, which, in turn, increases quiescence in cancer cells. They can also support cancer growth through the production of TGF-β2. CNS cells can produce IL1β and TNFα, which can push the astrocytes into a vicious cycle of overactivation. Pericytes produce IGF2 and chemokines, which support cancer metastasis. Cancer cells produce VEGFA, TGF-β, and PDGFB, which selectively increase a favorable pericytes phenotype. Fibroblasts constitute critical support for cancer cell growth through the production of MMPs and tissue growth factors. Different microglia types can either inhibit or support cancer cell growth. One of the strategies microglia can use to support tumor growth is inhibiting T cells. On the same note, dendritic cells can produce IDO and ICOSL, which recruit Tregs, which, in turn, inhibit pro-inflammatory CD4+ T cells, such as Th1 and Th17. Created with Biorender.com.

Astrocytes seem to play a dual role in cancer metastasis. Astrocytes were reported to support endothelial cells in resisting cancer cells infiltration. It has been reported that astrocytes secrete laminin-211 in the parenchymal basement membrane (Figure 6). Laminin-211 transmits pro-dormancy signals via dystroglycan and promotes quiescence by inhibiting yes-associated protein (YAP) in the cytoplasm of cancer cells. YAP is a well-known transcription regulator which plays an important role in cell proliferation. This process prevents the activation of growth-promoting mechanisms, which appears to be necessary for brain metastases [137]. However, there are various pathways through which astrocytes support cancer metastatic colonization. For example, extravasation of cancer cells penetrating the brain is associated with the astrocytes’ activation and upregulation of heparinase and matrix metalloproteinase-9, which promote further invasion and colonization of metastatic cells by degrading the ECM. Astrocytes can produce transforming growth factor-beta 2 (TGF-β2), which, in turn, regulates SMAD-mediated ANGPTL4 expression in cancer cells, contributing to successful colonization [138]. Astrocytes were demonstrated to support lung and breast cancer cells metastasis by producing interleukin-1 beta (IL1β) and tumor necrosis factor-alpha (TNFα) [139]. Xing et al. showed that brain metastases express high levels of IL1β under the influence of surrounding astrocytes, which leads to increased Notch signaling in cancer stem cells, promoting their stemness and growth in the metastatic niche [140]. In addition, IL1β and TNFα released by cancer cells increase TGF-β2 expression in astrocytes [141]. Evidence has also shown the formation of gap junctions between astrocytes and tumor cells, which allows for the passage of an intracellular second messenger 2′3′-cyclic GMP-AMP (cGAMP), which activates the STING pathway in astrocytes and promotes the expression of interferon α (IFNα) and TNFα to further facilitate tumor growth [142]. Other factors released by astrocytes that support cancer cell survival in the CNS include cytokines, stromal cell-derived factor 1 (SDF-1), sphingosine-1 phosphate, and glial-derived neurotrophic factor [143]. Astrocytes can protect tumor cells from chemotherapy by the sequestration of calcium from cancer cells and by regulating survival genes. The threshold for astrocytes switching sides is not yet known. However, in the case of breast cancer metastasis, it was shown that, along with the growth of metastatic lesions, astrocytes were gradually expelled to the border of the tumor [144]. We can speculate that the process is time specific and is cunningly regulated by cancer cells, not only to neutralize the astrocytes’ effect, but also to exploit their abilities for the metastatic cells’ benefit. At the early stages of brain invasion, cancer cells may release migratory signals that encourage astrocytes to free the space needed for tumor growth. After that, the production of pro-inflammatory cytokines, such as interleukin IL1β, can overactivate astrocytes. After the cancer cells trick the astrocytes into overactivation, astrocytes enter a vicious cycle of continuous overactivation by IL1β and TGF-β2.

Several opportunities might be available to target the described relationship, such as targeting migratory elements produced by the cancer cells in the early stages of brain metastasis. Reduction of inflammatory signals preventing astrocytes from being overactivated could also be a promising therapeutic strategy. However, experimental validation is still needed to support this hypothesis.

A significant role in supporting cancer metastasis in the brain seems to be fulfilled by pericytes. They produce chemokines that can attract cancer cells. Pericytes also secrete high volumes of extracellular matrix proteins, supporting the adhesion of melanoma and triple-negative cancer cells. Recently, it was discovered that pericytes produce insulin-like growth factor 2 (IGF2). IGF2 supports the proliferation efforts of mammary carcinoma but not melanoma cells. Inhibiting IGF2 signaling using silencing technology or picropodophyllin (PPP) decreased the size of brain tumors in mice inoculated with triple-negative breast cancer cells [145]. These results indicate that brain pericytes have significant pro-metastatic features, especially in breast cancer [145]. However, several questions remain unanswered. Why are cancer cells capable of exploiting pericytes to their advantage without any resistance on the pericytes’ side? It has been reported that tumor cells, through the production of PDGF-BB, lead to pericyte removal from the vessel and vessel sprouting. Tumor cells can also alter pericyte differentiation through the production of VEGF. Furthermore, cancer cells produce several growth factors, such as PDGF-B, VEGFA, and TGF-β1, which may trigger the prevalence of a PDGFR+/desmin+ pericyte phenotype and not the PDGFR+/CD13+ phenotype. Interestingly, PDGFR+/desmin+ pericytes, but not PDGFR+/CD13+, have been associated with the metastasis and proliferation of cancer cells. However, these findings highlight the importance of further investigations that aim to identify various pericyte subpopulations and compare their contribution to cancer metastasis. Pericyte subpopulations have been proposed to be highly plastic, which means that they can change their phenotype to other types of cellular lineage, such as microglial or vascular lineages. Thus, further investigations are needed to unravel the capabilities of pericyte subpopulation plasticity and how they influence metastasis [146].

Upon colonization, disseminating metastatic cells interact with and exploit the surroundings to support their growth, possibly through autocrine and paracrine loops [147]. For example, fibroblasts can increase metastases through the production of tissue growth factors that, in turn, can enhance tumor growth. Fibroblasts can also produce MMPs that can degrade the ECM. Additionally, mesenchymal stromal cells can produce TGF-β and soluble decoy T cell ligands that inhibit T cells’ interaction with tumor cells [148].

The microglial contribution to cancer metastasis is still obscure. There is evidence that supports a pro-metastatic role of the microglia [149]. However, there are various observations that draw a more complex picture. The tumor microenvironment is dominated by microglia and infiltrating macrophages. Microglia remain quiescent during normal homeostatic conditions but become activated in response to malignant cell infiltration [149]. Factors supporting the hypothesis that microglia support tumor growth include:

(i) Activation of microglia in the areas surrounding metastatic sites has been widely documented [149,150]. These activated microglia are characterized by increased anti-inflammatory cytokine production, decreased phagocytic activity, growth factor release, a chemo-attractive effect on peripheral monocytes, and inhibition of T cell proliferation within the tumor microenvironment. However, they exhibit the upregulation of proteins that support tumor cell adhesions such as: cellular adhesion molecules, CAMs, LFA-1, ALCAM, and E-selectin. It has been demonstrated that metastatic lung cancer cells produce IL6, which is able to induce anti-inflammatory action of microglia by switching JAK2/STAT3 signaling [151]. Furthermore, macrophages “cooperate” with cancer cells in a paracrinne manner, generating a positive feedback loop. Macrophages, by expressing EGF, promote protrusion formation in cancer cells, therefore enhancing cancer invasion. Simultaneously, cancer cells produce colony-stimulating factor-1 (CSF-1) that strenghtens EGF expression in macrophages. EGF in turn promotes CSF-1 expression in cancer cells [152];
(ii) In glioblastoma, the most aggressive form of solid, primary tumor of the CNS, microglia contribute significantly to the total tumor mass, indicating that they play an important role in tumor progression within the CNS.

However, recent findings have revealed that the microglia’s contribution to metastatic growth is affected by various other factors, including:

(i) Utilization of NO by microglia to induce tumor cell apoptosis. Inhibiting microglial activation using neurotrophin NT-3 is associated with an increase in brain metastasis formation [153,154];
(ii) The observation that microglia represent a heterogeneous combination of different groups of subpopulations that differ in their inflammatory profiles considerably. These subpopulations contribute differently to the tumor microenvironment [155]. Those immune cells with a pro-inflammatory phenotype have been described as exerting an antitumorigenic effect, while those with an anti-inflammatory profile have shown tumor-supporting activity. Furthermore, the exact role of certain microglial subpopulations is still not clear. For example, disease-associated microglia (DAM) deep transcriptomic analysis revealed an upregulated profile of several microglial genes such as Lgals3, Trem2, NOS2, and COX1 [156]. However, how these different pathways interact with the invading cells is yet to be determined;
(iii) Transition between pro- and anti-inflammatory states of the same subpopulation of microglia. The processes underlying the phenotypic switch between these two states and the potential coexistence of both identities during metastasis formation in the brain are poorly understood [157];
(iv) Comparability of the phenotypes of microglia and bone-marrow-derived macrophages (BMDMs) infiltrating the brain. Microglia develop from embryonic yolk sac progenitor cells and remain in the CNS, but bone-marrow-derived macrophages (BMDMs) penetrate the brain during metastasis [158,159]. Up to now, no unique markers that can distinguish between the activities of microglia and BMDM have been discovered.

Dendritic cells’ contribution to brain metastasis can be classified based on their subtype. Dendritic cells are in charge of starting adaptive immune reactions (including Treg recruitment). Interestingly, one group of dendritic cells, known as plasmacytoid dendritic cells (pDC), has been shown to play an important role in BM. It was shown that pDC produce IDO and ICSOL, which support Tregs recruitment [160]. Tregs are known to suppress Th1, Th2, and Th17 cells [161]. Thus, pDC could be viewed as supporting brain metastasis by supporting Tregs’ role in inhibiting the pro-inflammatory cells needed to disrupt the metastatic system. On the other hand, dendritic cells that have arisen from the myeloid linage are known to be pro-inflammatory and, thus, support antitumor immunity [162]. Additionally, it has been shown that tumor cells within the brain are capable of inhibiting the maturation of DC [163]. This prevents them from proper antigen presentation and enhances their immunosuppression activity by inducing the production of Il10 and TGF-β, hence, Treg recruitment [164].

Further investigation of tumor-infiltrating lymphocytes’ (TILs) interactions with metastatic tumors is still needed. During the process of tumor metastasis to the brain, the BBB becomes compromised, increasing its permeability. This, in turn, increases the number of immune cells accessing the brain and trying to infiltrate the metastatic brain tumor. Conversely, we found that number of CD4+ T cells infiltrating the brain increases during peripheral immune conditions, with no significant disintegration of the BBB [165]. Furthermore, CD4+ T cell infiltration of the brain could also be through areas lacking a BBB, such as the median eminence of the hypothalamus [165]. Once in the brain, TIL infiltration of the metastatic tumors has been suggested to follow one of three main mechanisms, namely, matrix infiltration, peritumoral infiltration, or diffuse infiltration. However, it is still not known whether these mechanisms are cancer type specific. Moreover, the molecular pathways controlling these mechanisms are still not well understood. Furthermore, it has been reported that the numbers of TILs (CD3+ T cells, CD8+ T cells, and CD45+ T cells) in brain metastases correlate positively with the prognosis [166]. However, this hypothesis overlooks the complexity within each T cell population. For example, CD4+ T cells are a heterogeneous group of cells composed of pro-inflammatory cells, such as Th1, and anti-inflammatory cells, such as Tregs, as well as cells such as Th17 that can switch between different inflammatory conditions [167,168,169]. CD4+ T cells are also highly plastic; thus, they can switch from one state to the other based on the presence of specific cytokines [170]. Furthermore, Tregs are composed of different subtypes that differ in their function and their immunosuppressive effect [161]. Our understanding of how these TILs function is based on their interaction with the primary tumor. However, there is a significant difference between the immune profiles of TILs infiltrating the primary cancer locations and the parenchyma of brain metastases. For example, it has been shown that TILs localized in brain metastases caused by lung cancer show stronger immunosuppressive abilities compared to the TILs isolated from the primary tumor. Furthermore, T cell receptor (TCR) repertoire comparison between T cells from metastasis regions and those of the primary regions showed a difference in abundance and diversity. Moreover, the interaction between B cells and the migrating metastatic cells has not been yet investigated, especially on the level of B cell subpopulations such as regulatory B cells (Bregs) [171].

## 4. Future Prospects and Conclusions

The journey of cancers cells from their primary location to the brain is still not fully mapped. We know that cancer cells follow a typical route starting from EMT, passing through infiltration of the ECM, blood vessels, and the BBB, and ending up with angiogenesis and colonization. However, our review leaves several questions unanswered. For example, there could be unknown factors lying behind the choice of the intravasatiion route (e.g., blood vessel vs. lymphatic vessel). Notably, the choices behind the cancer cells’ final destination within the brain regions are also not known. Similarly, our review highlights the need to better understand the interaction of cancers cells with their microenvironment in the brain at a single-cell level. Several attempts have been initiated to investigate the interaction between microglia and cancer cells using single-cell analyses [172]. However, less is known about the interaction between cancer cells and the rest of immune cells in the brain. Understanding these relationships will shed more light on the possible molecular candidates that could be therapeutic targets for halting cancer progression, including metastases formation in the brain.

## Figures and Tables

**Table 1:** Primary cancer types grouped by rate of brain metastasis and rate of survival (adopted from [2]).

Cancer Type	Rate of Brain Metastasis (%)	Rate of Survival by Month
Colorectal cancer	0.27	3–17
Breast	0.41	3–36
Lung	12	7–46
Kidney	1.48	4–35
Melanoma	0.65	5–34
